# Representations of depression and schizophrenia in the community: The role of illness and risk perceptions on help-seeking intentions

**DOI:** 10.3389/fpsyg.2022.1011195

**Published:** 2022-11-24

**Authors:** David Dias Neto, Maria João Figueiras, Rita Sebastião

**Affiliations:** ^1^School of Psychology, ISPA-Instituto Universitário, Lisbon, Portugal; ^2^Applied Psychology Research Centre Capabilities and Inclusion, Lisbon, Portugal; ^3^Department of Psychology, College of Natural and Health Sciences, Zayed University, Abu Dhabi, United Arab Emirates

**Keywords:** illness perceptions, risk perceptions, help-seeking, depression, schizophrenia

## Abstract

**Objective:**

Illness perceptions (IPs) are important in understanding human reactions to illnesses, including mental health disorders. They influence risk perceptions and several variables relevant to the adjustment to a disorder, treatment seeking, and health outcomes. This study sought to compare IP, risk perception, and help-seeking intention for depression and schizophrenia in a community sample and to assess the mediating role of risk perception in the relationship between IP and help-seeking intention.

**Materials and methods:**

A total of 380 adults participated in this study and filled out self-report measures of IPs, risk perceptions, and help-seeking intention. The previous diagnosis of depression was used to control the comparisons between the two disorders. A structural equation model (SEM) was used to test the mediation relationship.

**Results:**

Perceived consequences, expected timeline, lack of personal control, and symptom identity were higher for schizophrenia, while lack of treatment control and concern were higher for depression. An interaction occurred with a previous diagnosis of depression for several dimensions of IP. Concerning the SEM, a valid model was obtained for depression, explaining 15.5% of help-seeking intentions, but not for schizophrenia.

**Conclusion:**

The results show that the general population represents depression and schizophrenia differently. These representations are influenced by having experienced depression, and that illness and risk perceptions contribute to explaining the intention to seek help. Considering these illness representations makes it possible to understand the general population’s emotional and cognitive reactions to mental health disorders.

## Introduction

Mental health and mental illness representations are essential in understanding a wide range of phenomena, such as health literacy, stigma, self-stigma, treatment adherence, or even distress associated with the mental health disorder. Studies have shown that the representation of personal illness relates to self-stigma and measures of the disorder itself, for example, in schizophrenia (Chuang et al., 2019). To understand these results, it is useful to consider models of health representation. These models have been proposed for general health and have been applied to a significant number of conditions.

One of the most important models of illness representation is the common-sense model of self-regulation developed by Diefenbach and Leventhal (1996) and Martin et al. (2003). According to this model, illness representations or perceptions (IPs) are grouped into different but interrelated components. These components have been classified as cognitive or emotional illness representations (Broadbent et al., 2006). The emotional IP includes: (a) concern about the disease or its consequences and (b) the emotional response (e.g., anger, fear, and distress) associated with the illness. The cognitive IPs include representations about: (c) the consequences of a particular illness, (d) the expected timeline or duration of the illness, (e) personal control of aspects of the disease, (f) the extent of the usefulness of treatment in controlling or managing the illness, (g) the perception of the experience of an illness, and (h) its symptoms and understanding or being knowledgeable of the disease.

With decades of research, IP has been related to several outcomes and physical illnesses, supporting the concept’s relevance (Kaptein et al., 2015). According to Weinman and Petrie (1997), the way a patient understands their illness can help to understand their behavior and new ways of adjusting to illness. Given the wealth of data, research has been conducted on the mechanisms through which IP operate. IPs may influence outcomes on themselves or through other processes such as coping (Dempster et al., 2015) or risk perceptions (Figueiras et al., 2022a). Risk perception, in particular, is a promising factor as it may link illness representations with outcomes. General IPs are considered to be the basis for risk perception. This is supported by several studies that have tried to relate them and study their relationship with outcomes (Freeman-Gibb et al., 2017; Figueiras et al., 2022b). Given the existence of inconsistent results, further research is needed to explore this relationship (Peleg et al., 2016).

The research on the relationships between IP and mental health has primarily been conducted on patients with physical health issues. Several mental health-related outcomes have been found to be related to the IP of patients with physical conditions. This link is considered using general measures of symptoms (Hamama and Levin-Dagan, 2022), depression (Foxwell et al., 2013; Kaptein et al., 2015; de Heer et al., 2017), and stress (Karademas et al., 2009; Westbrook et al., 2016; Zhang et al., 2016).

More recently, there has been a growing interest in using the common-sense model of self-regulation in the context of mental illness (Antoniades et al., 2017; Scerri et al., 2019). Some early studies found that IPs were associated with quality of life and coping in schizophrenia, with worse consequences scores found to be the strongest predictor of poor functioning and quality of life (Lobban et al., 2004). Similarly, in IPs in patients with non-affective psychotic disorder, the identity dimension strongly predicted depression, anxiety, and self-esteem (Watson et al., 2006). In high users of mental health services, negative IPs were associated with high unmet needs, low functioning, and a poor attitude toward medication and doctor visits (Broadbent et al., 2008). Another study assessed IPs and health beliefs in people with common mental disorders (e.g., depression and anxiety disorders) and found a significant correlation with attitudes toward seeking professional psychological help (Goyal et al., 2020).

The first systematic review examined 13 studies (Baines and Wittkowski, 2013) of patients with psychosis, bipolar disorder, depression, and eating disorders using the illness perception questionnaire (IPQ and IPQ-R). The results showed that mental health is perceived as cyclical, with severe consequences, and chronic. Several health outcomes were related to different IPs. Help-seeking behavior and coping were related to perceptions of chronicity, controllability, and negative consequences. Engagement with services and health seeking were associated with perceptions of illness coherence, and finally, treatment adherence was related to positive beliefs about treatment. Despite most of the studies being cross-sectional, the results were in accordance with the common-sense model of self-regulation. Wong et al. (2018) conducted a systematic review of the existing studies of IPs in children with attention deficit hyperactivity disorder (ADHD) and their parents. The results of the 101 studies show a closer attention in the literature to the issue of perceived effectiveness of treatment relative to other dimensions of IP. The different studies also showed relevant variability in the scores of IP, suggesting the need to study moderators and relevant predictors. In any case, the research points to a promising role of IP in understanding coping strategies, treatment adherence, and wellbeing. A recent review (Averous et al., 2020) showed that illness representations relate to psychosocial adjustment and treatment outcomes in people with mental disorders. More specifically, in adulthood, illness representations are associated with adherence to medication and psychotherapies [for bipolar disorders, psychotic disorders, depression, mixed mental disorders, and post-traumatic stress disorder (PTSD)] and with the individual’s attitude toward medications, such as the need to take them, their harmfulness, as well as distrust of medicine (for psychotic disorders, depression, and mixed mental disorders).

Fewer studies have examined the role of IPs and risk perceptions for mental health in the community or non-clinical samples. Warner et al. (2020) studied risk perception in a sample of Canadians at risk for depression. Risk perception and other socio-demographic variables were associated with self-help behaviors. There was a different profile according to gender. In a sample of older Japanese individuals, risk perceptions were not associated with depression levels but were linked to perceived knowledge about depression (Imai et al., 2015). In gamblers, risk perceptions have been associated with disordered gambling, which is explained by its effect on decision-making (Spurrier and Blaszczynski, 2014). In community samples, the IP of relevant conditions also correlates with mental health outcomes. For example, during the COVID-19 pandemic, IPs were associated with general stress and other measures (Gloster et al., 2020; Han et al., 2021; Neto et al., 2021). For breast cancer, IP has been linked to breast cancer worry in healthy women (Gibbons and Groarke, 2016).

The literature suggests that illness and risk perceptions play an important role in the psychological reaction to mental health conditions in both patients and community dwellers. To further our understanding of the role of IP in mental health, it is essential to study the differences in how different conditions are represented in the community and what the mechanisms are that translate mental IP into particular outcomes. The present study sought to compare IP of depression and schizophrenia in a community sample. These two disorders were chosen considering the differences in presentation and prevalence (American Psychiatric Association [APA], 2013). They have two different prevalence profiles for gender and age, showing a different course and functional impact.

Furthermore, they are subject to different levels of stigma (Wood et al., 2014), which may relate to different personal representations. Both the epidemiological and social differences make these two disorders sufficiently different to study the contrasts in IP. To address the issue of the mechanisms through which IP operates, a particular model for the anticipated relationships was tested. Based on the common-sense self-regulation theory, this model explores the relationship between illness and risk perceptions in affecting help-seeking intentions.

The goals of the present research are: (a) to compare IPs, risk perceptions, and help-seeking intentions for depression and schizophrenia; (b) to relate risk perception with gender and age; and (c) to assess the mediating role of risk perception in the relationship between IP and help-seeking intention. Considering the different prevalence’s of depression and schizophrenia, it is possible that a history of depression (i.e., the disorder’s experience) may influence IP differently. For this reason, past diagnoses of depression were controlled for in the comparison in goal (a). The relations studied in goal (c) explore the relationships between illness and risk perceptions in explaining behavioral intentions, thus expanding the common-sense model of self-regulation. Understanding how these disorders are perceived is essential to understanding stigma and mental health literacy processes. These are crucial in addressing difficulties and inequalities in seeking help for mental health conditions (Neto et al., 2017).

## Materials and methods

### Participants

This study examines a convenience sample of the general population. The sample comprised 380 adult participants with the ability to read and write and use the technologies involved in the online survey. Participants were mostly female (315, 82.9%) with an average age of 49.2 (SD = 13.93, Min. = 18, and Max. = 84). The majority were married or in a civil union (191, 50.3%) or single (99, 26.1%), with a typical education level for Portugal, mostly BA (147, 38.7%) or secondary school (107, 28.2%). The sample was fairly distributed across the country, with the most frequent districts of residence being Lisboa (141, 37.1%), Porto (65, 17.1%), and Setúbal (43, 11.3%).

A relevant proportion of participants reported having been diagnosed, in the past, with a mental health disorder by a health professional. A total of 200 participants reported having had depression (52.6%), and three reported having had schizophrenia (0.8%). The results for depression are somewhat higher than expected, even considering the high prevalence of depression in Portugal (Almeida and Xavier, 2013). A contributing factor to explaining this was the COVID pandemic, which has increased the incidence of mental health disorders (Gloster et al., 2020).

### Instruments

A questionnaire was included to assess socio-demographic variables. It also included two questions to evaluate a past diagnosis of depression and schizophrenia by a health professional. Participants were asked to respond dichotomously (i.e., yes or no).

#### Illness perceptions

The Brief IPQ (B-IPQ) was used to assess the IPs as proposed in the common-sense model of self-regulation (Weinman and Petrie, 1997). The B-IPQ is a reduced version of the revised IPQ for a specific disease. It uses eight questions, each representing a dimension of disease perception: consequences, timeline, personal control, treatment control, identity, concern, understanding, and emotional response. There are three reversed items (personal control, treatment control, and understanding). Each question is answered on a semantic differential scale, ranging from 0 to 10, on the importance of each dimension for the patient, with higher scores reflecting more negative IPs. The B-IPQ does not allow for a total score considering that each IP is considered independently, despite different authors having used different composite scores in the literature. This instrument has been widely used and has shown good psychometric properties (Broadbent et al., 2006; Figueiras et al., 2010).

#### Risk perceptions

Risk perceptions were assessed by two items asking for estimates of perceived personal risk (i.e., how likely do you feel at risk of developing depression in the next 10 years?) and compared risk (i.e., considering someone of the same age as you, how likely do you feel at risk of developing depression in the next 10 years?). These items were used and analyzed in previous studies on risk perception in non-clinical samples (Figueiras et al., 2022a,b). The participants were asked to rate the perceived individual and compared the risk for each disorder by rating it on an 11-point scale ranging from 0 (no risk) to 10 (high risk). Originally, the scale was inspired by previous literature on verbal probability expressions used to communicate risk (Cameron and Reeve, 2006) and the research on the correlates of perceived susceptibility (Gerend et al., 2004).

#### Intention to seek help

The intention to seek help was assessed by three items referring to the type of help considered: medication (i.e., what is the likelihood of accepting medication, if prescribed by a medical doctor, if you developed depression/schizophrenia?), psychological intervention [i.e., what is the likelihood of seeking psychological intervention (e.g., psychotherapy) if you developed depression/schizophrenia?], and self-help [i.e., what is the likelihood doing activities promoting wellbeing (e.g., meditation, exercise, and self-help books) if you developed depression/schizophrenia?]. Like in the previous variables, the same semantic differentiation scale of 11 points was used (from very unlikely to very likely).

### Procedure

The Ethical Committee of ISPA–Instituto Universitário (Ref.: I-091-7-22) approved the study. All participants signaled their informed consent before completing the survey, which was anonymous and voluntary. The survey was implemented on Qualtrics, and the order of the instruments was as follows: B-IPQ, risk perceptions, intention to seek help, and socio-demographic variables. The participants first filled out the questionnaires concerning depression, followed by the same instruments for schizophrenia. The choice of addressing depression first was based on the fact that schizophrenia has a greater stigma in the community and could bias the assessment of depression IP. The study was disseminated *via* social networks using paid advertisements and specific groups not related to mental health (e.g., neighbors groups). Following open science practices (Vicente-Saez and Martinez-Fuentes, 2018), the anonymized database and syntaxes are made available freely (see link on sharing statement). Unlike such recommendations, hypotheses were not registered in advance. Data were gathered from March to June 2022.

### Analysis

A mixed analysis of variance (ANOVA) was used to test the effect of the repeated-measure factor diagnosis (i.e., depression vs. schizophrenia) and the independent factor of the previous diagnosis of depression (i.e., having or not having had depression) on each of the dependent variables and interaction effects. Participants (*n* = 19) who reported not knowing or preferring not to tell were excluded from all the analyses that implied considering the previous diagnosis. To study the second goal, *t*-tests and Pearson’s correlations were used. These analyses were conducted using SPSS (IBM Corp, 2021). The significance level considered was 0.05.

The study of the mediating role of risk perceptions in the relationship between IP and intention to seek help was conducted with structural equation models (SEMs). Two models were tested for depression and schizophrenia. Items with factor loadings < 0.5 were removed from the model (Maroco, 2021). They were fitted with the lavaan package (Rosseel, 2012) for the R statistical system. Regarding depression, estimation was carried out using maximum likelihood estimation. The goodness-of-fit for the model was assessed through the Chi-square statistics, Comparative Fit Index (CFI), Tucker–Lewis Index (TLI), Root Mean Square Error of Approximation (RMSEA), and Standardized Root Mean Squared Residual (SRMR). For schizophrenia, the estimation was carried out using robust maximum likelihood estimation since several observed variables deviated from the normal distribution (skewness ranged from −3.850 to −0.410; kurtosis ranged from 18.833 to −0.104). The goodness-of-fit for this model was assessed through the robust versions of these indices. The fit was judged adequate for CFI and TLI above 0.9 and RMSEA and SRMR below 0.08 (Maroco, 2021).

## Results

### Illness representations of depression and schizophrenia

Each IP, risk perception, and intention to seek help were compared considering the diagnosis (depression vs. schizophrenia) and previous diagnosis of depression. Table 1 presents the main effects of the disorder and interaction effects. Concerning IP, a strong main effect of diagnosis was found for timeline, concern, understanding, and emotional response. A medium effect was found for consequences and personal control, and a small effect was found for treatment control and identity. Regarding risk perceptions, there was a strong main effect of diagnosis on both personal and compared risk. Concerning the intention to seek help, the only significant effect (moderate in size) was for medication. Week-to-moderate interaction effects were found for four IPs (timeline, treatment control, understanding, and emotional response) for risk perceptions and intention to seek help using medication.

**TABLE 1:** Main effects of the disorder type and interaction effects–analysis of variance (ANOVA) [*n* = 361, *F*(1, 359)].

VD	Factor	*F*	*p*	Partial η^2^
**Illness perceptions**				
Consequences	Disorder	36.543	< 0.001	0.092
	Disorder × Past diagnosis	0.779	0.378	0.002
Timeline	Disorder	411.415	< 0.001	0.534
	Disorder × Past diagnosis	13.905	< 0.001	0.037
Personal control*	Disorder	32.396	< 0.001	0.083
	Disorder × Past diagnosis	2.331	0.128	0.006
Treatment control*	Disorder	8.746	0.003	0.024
	Disorder × Past diagnosis	5.448	0.020	0.015
Identity	Disorder	19.334	< 0.001	0.051
	Disorder × Past diagnosis	0.194	0.660	0.001
Concern	Disorder	79.920	< 0.001	0.182
	Disorder × Past diagnosis	0.839	0.360	0.002
Understanding*	Disorder	166.371	< 0.001	0.317
	Disorder × Past diagnosis	35.512	< 0.001	0.090
Emotional response	Disorder	56.421	< 0.001	0.136
	Disorder × Past diagnosis	4.568	0.033	0.013
**Risk perceptions**				
Personal	Disorder	556.426	< 0.001	0.608
	Disorder × Past diagnosis	30.910	< 0.001	0.079
Compared	Disorder	557.997	< 0.001	0.609
	Disorder × Past diagnosis	36.561	< 0.001	0.092
**Help-seeking intentions**				
Medication	Disorder	24.691	< 0.001	0.064
	Disorder × Past diagnosis	9.092	0.003	0.025
Psychological	Disorder	0.363	0.547	0.001
	Disorder × Past diagnosis	0.863	0.354	0.002
Self-help	Disorder	0.498	0.481	0.001
	Disorder × Past diagnosis	2.899	0.089	0.008

The table presents the main effect of the disorders (depression and schizophrenia) and the interaction of the Disorder × Past diagnosis (i.e., having been diagnosed with depression).

*Reverse score items (higher values reflect more negative perceptions).

Table 2 shows the means and standard deviations for all groups. In general, the IPs are negative except for treatment control and understanding. The following IPs are more negative for schizophrenia: consequences, timeline, personal control, and identity. Conversely, the following IP are more negative for depression: treatment control and concern. Both risk perceptions were higher for depression, which was on the likely side of the item’s scale, unlike schizophrenia. There was a positive intention to seek help in all categories, with the intention to seek medication being higher for schizophrenia relative to depression.

**TABLE 2:** Means and SD for depression or schizophrenia considering past diagnosis (*n* = 361).

		Past diagnosis		Without diagnosis	
		(*N* = 200)		(*N* = 161)	
		
		*M*	SD	*M*	SD
**Illness perceptions**					
Consequences	Depression	8.9	1.77	8.4	2.34
	Schizophrenia	9.5	1.37	9.2	1.68
Timeline	Depression	8.0	1.78	7.0	1.85
	Schizophrenia	9.7	0.87	9.5	1.41
Personal control*	Depression	6.7	2.31	6.2	2.17
	Schizophrenia	7.3	3.05	7.3	2.50
Treatment control*	Depression	1.7	2.03	1.4	1.96
	Schizophrenia	1.1	1.79	1.4	1.89
Identity	Depression	7.3	2.09	6.9	2.08
	Schizophrenia	7.9	2.31	7.4	2.46
Concern	Depression	8.5	2.08	7.3	2.60
	Schizophrenia	6.9	3.46	6.0	3.39
Understanding*	Depression	1.2	1.75	2.9	2.29
	Schizophrenia	3.8	3.01	3.9	2.92
Emotional response	Depression	7.3	2.81	5.4	3.01
	Schizophrenia	5.6	3.39	4.5	3.40
**Risk perceptions**					
Personal	Depression	7.5	2.73	4.7	2.53
	Schizophrenia	2.7	2.98	1.7	2.10
Compared	Depression	7.5	2.66	4.6	2.59
	Schizophrenia	2.7	2.97	1.7	2.17
**Help-seeking intentions**					
Medication	Depression	8.1	2.71	7.2	3.21
	Schizophrenia	8.4	2.82	8.6	2.52
Psychological	Depression	8.6	2.09	8.4	2.54
	Schizophrenia	8.6	2.60	8.6	2.37
Self-help	Depression	8.2	2.37	8.5	2.15
	Schizophrenia	7.9	3.02	8.6	2.30

*Reverse score items (higher values reflect more negative perceptions).

Considering the significant interaction effects concerning the IP, namely, timeline, treatment control, and emotional response, more negative scores for depression in participants with a previous diagnosis of depression. In the opposite direction, understanding showed higher negative scores for depression in participants without a prior diagnosis of depression. Both risk perceptions were more negative for depression in participants with a previous diagnosis of depression. In the same direction, the intention to take medication was higher for depression in participants with a prior diagnosis of depression.

### Differences in risk perception and intention to seek help according to age and gender

Age showed no significant correlations with risk perception and most items on the intention to seek help for depression and schizophrenia. The only exception was the intention to take medication for depression, *r*(380) = 0.20, *p* < 0.001, which showed a weak positive association. Table 3 shows the differences concerning gender. Female participants showed higher personal and compared risk (*M* = 6.4, SD = 2.93 and *M* = 6.4, SD = 2.95) than male (*M* = 5.5, SD = 3.13 and *M* = 5.4, SD = 3.00) for depression. Concerning intention to seek help for depression, female participants reported higher intention to take medication (*M* = 7.7, SD = 2.94) or seek psychological intervention (*M* = 8.6, SD = 2.23) relative to male participants (*M* = 6.8, SD = 3.27 and *M* = 7.7, SD = 2.58). For schizophrenia, female participants were more likely to seek psychological intervention (*M* = 8.7, SD = 2.38) than male participants (*M* = 7.8, SD = 2.77).

**TABLE 3:** Gender differences in risk perception and intention to seek help (*df* = 378).

	Depression			Schizophrenia		
	*t*-test	*p*	Cohen’s *d*	*t*-test	*p*	Cohen’s *d*
**Risk perceptions**						
Personal	2.382	0.018	0.324	–1.463	0.144	–0.199
Compared	2.253	0.012	0.344	–1.498	0.135	–0.204
**Help-seeking intentions**						
Medication	2.353	0.019	0.321	1.711	0.088	0.233
Psychological	2.979	0.003	0.406	2.679	0.008	0.365
Self-help	0.644	0.520	0.088	0.591	0.555	0.080

### Relationship between illness perceptions, risk perception, and intention to seek help

Two models assessing the mediation role of risk perception in the relationship between IP and intention to seek help were tested for schizophrenia and depression. Regarding schizophrenia, the proposed model showed a poor model fit to the data: χ^2^(40) = 136.097, *p* < 0.001, CFI = 0.939, TLI = 0.916, RMSEA = 0.080, *p* = 0.001, and SRMR = 0.093. No modification indices were used to improve fit, but the variance of emotional representations was constrained to 0.1.

**FIGURE 1:**
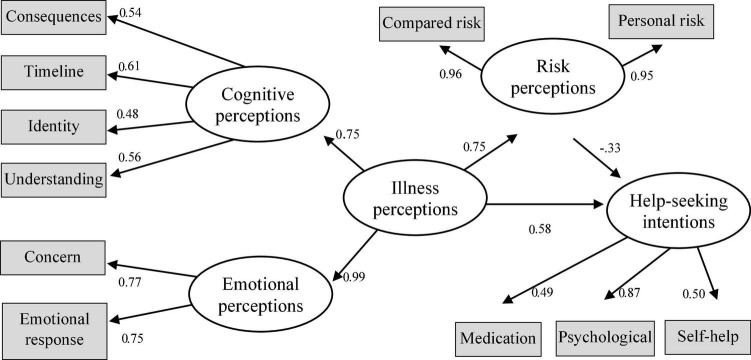
Structural equation model (SEM) on the role of illness perception (IP) and risk perceptions in help-seeking intentions for depression.

Regarding depression, the proposed model showed a good fit to the data: χ^2^(40) = 100.683, *p* < 0.001, CFI = 0.961, TLI = 0.946, RMSEA = 0.063, *p* = 0.076, and SRMR = 0.060. No modification indices were used to improve fit, but the variance of emotional representations was constrained to 0.1. Overall, the model explained 15.5% of the intention of seeking help and 56.4% of the risk perception. IPs had a standardized direct effect of 0.577 (*p* < 0.001) and a standardized indirect effect mediated by the risk perception of −0.251 (*p* = 0.006). The total standardized effect of IPs on the intention of seeking help was 0.325 (*p* < 0.001). This constitutes a suppression effect (MacKinnon et al., 2000), given that the indirect effect differed in valence from the direct effect, reducing the overall effect that was nevertheless significant. Figure 1 displays the standardized estimates for the depression help intention model.

## Discussion

The results of the study suggest that, as in physical conditions, IPs are promising in explaining mental health phenomena. They are discriminative enough to differentiate depression from schizophrenia in the general population, even considering the familiarity associated with having had depression. Not surprisingly, schizophrenia is thought to have more negative consequences, lasting longer, with the experience of more negative symptoms and less control. On the contrary, participants believe that they have less control over the treatment and are more concerned with depression. In general, participants are more likely to perceive a risk of having depression, both personally and when compared to a similar other, and are willing to seek help for both conditions. Concerning medication, participants are more likely to accept it if it were prescribed for schizophrenia relative to depression.

This study suggests that having or having had depression is linked with these perceptions by aggravating the judgments about depression–for timeline, treatment control, and emotional reaction–and by reducing the negativity on the perceptions of the experience of symptoms and increasing the sense of understanding. Having had depression is associated with participants being more aware of the negative consequences of depression, but may be linked with a higher sense of understanding of the disorder and tolerance to the experience of symptoms.

While these disorders have an epidemiological risk profile different for age and gender, the illness representations and intention to seek help only partially reflect this. Epidemiologically, depression is more frequent in women than men, and its first episode is highly distributed across ages. At the same time, schizophrenia is more frequent in men and most frequently initiates in the patient’s 20’s (American Psychiatric Association [APA], 2013). Regarding age, no associations with risk are found, and only a moderate association occurs between age and the likelihood of accepting medication prescribed for depression. Gender shows a greater consonance with epidemiological reality, with women being more likely to perceive the risk of having depression. This result agrees with what was found in the literature on risk perceptions of depression in non-clinical samples (Imai et al., 2015; Warner et al., 2020). Furthermore, women are more likely than men to intend to seek help for depression (medication and psychological intervention) and schizophrenia (psychological intervention). These results suggest that greater care should be given to public health policy to groups defined by age (e.g., older citizens at risk for depression) or to male citizens.

Concerning the tested model for depression, several observations deserve reflection. First, a valid model explaining 15.5% of the variation in the intention to seek help was found. IP predicted the intention to seek help, and a partial mediation was found for risk perceptions. This means that the way individuals represent depression influences the intention to seek help, and this relationship is influenced by the perception of risk. The nature of this influence was more complex than anticipated, given that a suppression effect (MacKinnon et al., 2000) of risk perception was found. A suppression effect happens when the indirect effect is different in valence, thus reducing the main effect. In this model, the effect of risk perception on the intention to seek help was negative. This means that while a negative IP predisposes participants to seek help, a higher risk has the opposite effect. A possible explanation is that the perception of higher risk activates avoidance or denial processes that negatively influence the intention to seek help. The perception of elevated risk may constitute an aversive state, which could promote avoidance. Future studies may shed light on this effect by comparing individuals with or without a disorder or comparing groups at the extremes of illness or risk perceptions.

A third observation is that, unlike depression, a valid model could not be found for schizophrenia. This means that the considered variables do not show multivariate relations in a manner that could be anticipated by the common-sense model of self-regulation (Diefenbach and Leventhal, 1996; Martin et al., 2003). Schizophrenia and depression may have different meanings for the participants. In accordance with epidemiological data (American Psychiatric Association [APA], 2013), while depression is perceived as a disorder that may occur in the future, schizophrenia is considered very unlikely. For schizophrenia, the judgment on the intention to seek help is, therefore, less linked to an actual possibility when compared to depression. The role of familiarity was already suggested on the effect of the previous history of depression in comparing depression and schizophrenia representations. Future research may further shed light on the role of familiarity and personal relevance in the effects of IP on outcomes.

Concerning theoretical considerations, this study further supports the usefulness of the common-sense model of self-regulation (Diefenbach and Leventhal, 1996; Martin et al., 2003). However, while the results suggest that IPs predictably affect help-seeking, this influence seems to be affected by the disorder type and familiarity. Furthermore, risk perception may be one process through which IP affects help-seeking. Longitudinal and experimental studies are needed to assess the eventual causal nature of these relationships.

This study has several limitations. The convenience nature of the sample may have brought unanticipated effects. The sample had a higher proportion of individuals who had depression, due to the participant gathering procedures or circumstantial events like the COVID pandemic. The self-reported nature of the variables may raise validity issues concerning variables such as a previous diagnosis of depression by a health professional. The order of the application of the instruments was the same for all participants. This may have produced order effects, namely in assessing the IP of depression and schizophrenia. Furthermore, the intention to seek help may not reflect actual behavior despite being an important indication. Finally, the sample size did not allow further analysis that could elucidate some of these results, such as comparing the tested model for participants with and without a history of depression.

Even considering these limitations, these results show the promising role of IP and risk perceptions in mental health. They are sensitive enough to be discriminative between disorders and be affected by the familiarity of the conditions. Given their relation to help-seeking intention, these perceptions may be important targets of public health interventions for the general public. Considering the high prevalence of mental health disorders and the existing barriers to help-seeking, understanding how our communities perceive these conditions is a crucial step.

## Data availability statement

The datasets and syntaxes are made available in https://osf.io/2e9sz/?view_only=a1a2a5c3da3443f8b963659d3b6b8b42.

## Ethics statement

The studies involving human participants were reviewed and approved by the Ethical Committee of ISPA–Instituto Universitário (Ref.: I-091-7-22). The patients/participants provided their written informed consent to participate in this study.

## Author contributions

DN and MF: design of the study, discussing the results, and writing up. DN and RS: literature review, data analysis, and data gathering. All authors reviewed and amended the manuscript, contributed to the article and approved the submitted version.
