# Corrigendum: Representations of depression and schizophrenia in the community: The role of illness and risk perceptions on help-seeking intentions

**DOI:** 10.3389/fpsyg.2022.1127367

**Published:** 2023-01-17

**Authors:** David Dias Neto, Maria João Figueiras, Rita Sebastião

**Affiliations:** ^1^School of Psychology, ISPA-Instituto Universitário, Lisbon, Portugal; ^2^Applied Psychology Research Centre Capabilities and Inclusion, Lisbon, Portugal; ^3^Department of Psychology, College of Natural and Health Sciences, Zayed University, Abu Dhabi, United Arab Emirates

**Keywords:** illness perceptions, risk perceptions, help-seeking, depression, schizophrenia

In the published article, there was an error in the Funding statement. The reference to the funding was incomplete. The correct Funding statement appears below.

